# Role of the Deep Eutectic Solvent Reline in the Synthesis of Gold Nanoparticles

**DOI:** 10.1021/acssuschemeng.2c07337

**Published:** 2023-07-03

**Authors:** Sukanya Datta, Julien Mahin, Emanuela Liberti, Iva Manasi, Karen J. Edler, Laura Torrente-Murciano

**Affiliations:** † Department of Chemical Engineering and Biotechnology, University of Cambridge, Philippa Fawcett Drive, CB3 0AS Cambridge, U.K.; ‡ Department of Materials, University of Oxford, OX1 3PH Oxford U.K.; § The Rosalind Franklin Institute, Harwell Science & Innovation Campus, Didcot, OX11 0QS Oxfordshire, U.K.; ∥ Department of Chemistry, University of Bath, Claverton Down Road, BA2 7AY Bath, U.K.

**Keywords:** gold nanoparticles, deep eutectic solvents, reline, gold speciation, sustainable solvents

## Abstract

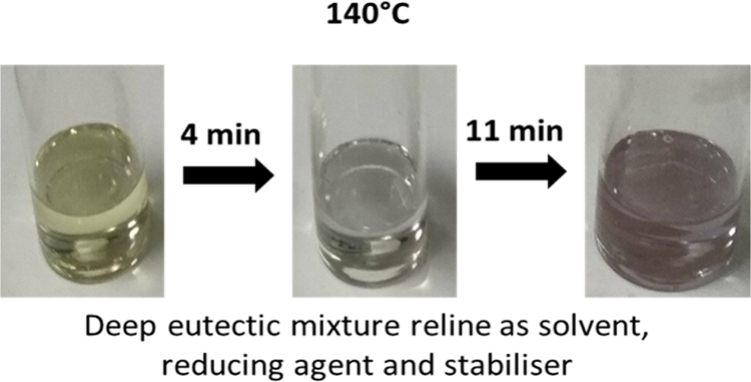

This work presents a mechanistic understanding of the synthesis of small (<3 nm) gold nanoparticles in a nontoxic, eco-friendly, and biodegradable eutectic mixture of choline chloride and urea (reline) without the addition of external reducing or stabilization agents. Reline acts as a reducing agent by releasing ammonia (*via* urea hydrolysis), forming gold nanoparticles even at trace ammonia concentration levels. Reline also affects the speciation of the gold precursor forming gold chloro-complexes, stabilizing Au^+^ species, leading to an easier reduction and avoiding the otherwise fast disproportionation reaction. Such a capability is however lost in the presence of large amounts of water, where water replaces the chloride ligands in the precursor speciation. In addition, reline acts as a weak stabilizing agent, leading to small particles (<3 nm) and narrow distributions although agglomerates quickly form. Such properties are maintained in the presence of water, indicating that it is linked to the urea stabilization rather than the hydrogen-bonding network. This work has important implications in the field of green synthesis of nanoparticles with small sizes, especially for biomedical and health care applications, due to the nontoxic nature of the components of deep eutectic solvents in contrast to the conventional routes.

## Abstract 2

Nontoxic, eco-friendly, and biodegradable eutectic mixtures can replace external reducing and stabilization agents in the synthesis of metal nanoparticles.

## Introduction

Gold nanoparticles present exceptional physical and chemical properties with a wide range of applications in catalysis, photonics, health care, and biomedicine among others.^1−5^ Such unique properties are strongly dependent on the size of nanoparticles, particularly in the case of particles with sizes < 10 nm, because of the low coordination and d states closer to the Fermi level of the surface gold atoms.^6^ However, development of such small metal nanoparticles is a key challenge in the field due to their tendency to agglomerate to reduce their surface energy.^7^

Wet methods such as chemical reduction are conventionally used for the synthesis of metal nanoparticles due to their easy scalability in comparison to physical methods. Such methods generally involve the reduction of a metal precursor (usually a metal salt) with a reducing agent in the presence of a stabilizer (via electrostatic or steric stabilization).^8^ Numerous mechanistic studies have demonstrated the link between fast nucleation rates (normally achieved using strong reducing agents such as sodium borohydride) and small sizes.^9^ Other conventional reduction routes such as the Turkevich method using relatively mild reducing agents lead to gold particles with sizes >10 nm.^10^

In the last few years, a range of deep eutectic solvents (DES), formed by the combination of Lewis or Brønsted acids or bases,^11,12^ have been used in the synthesis of nanomaterials as they are considered a green, eco-friendly subclass of ionic liquids.^13^ In particular, DES produced from a hydrogen bond donor and a quaternary ammonium salt offers an inexpensive, environmentally friendly, and nontoxic route to prepare nanomaterials with a small environmental footprint.^14^ Choline chloride-based DESs are the most popular ones due to their excellent solubilization of many metals.^15^ Within these DESs, reline, composed of a eutectic molar mixture (1:2) of choline chloride (m.p.: 303 °C) and urea (m.p.: 133 °C) with a resulting melting point in the range of 12–24 °C,^16^ has been widely explored. Our group has reported the role of reline as a supramolecular catalyst and a structure-directing agent in the synthesis of nanostructured ceria^14^ and vanadium oxide^17^ by providing less-energy-intensive pathways, while avoiding the use of highly concentrated base solutions by solvent-driven pre-organization of the precursors. Similarly, a number of studies have been reported on the wet chemistry synthesis of gold nanoparticles in reline by adding external reducing agents.^18−21^ Sun et al.,^18^ Stassi et al.,^21^ and Luna-Bárcenas et al.^19^ synthesized multiple twinned gold nanostructures such as stars and snowflakes by reducing HAuCl_4_ in reline using ascorbic acid as a reducing agent. They investigated the effect of different reaction parameters such as amount of water, reactant concentration, and temperature on the resulting gold nanostructures. In these studies, ascorbic acid acts both as a reducing agent and an anisotropic template directing agent due to its preferential adsorption on particular crystalline facets.^22^ The presence of water in the system accelerates the synthesis of gold nanoparticles because of the deprotonation of ascorbic acid to reduce HAuCl_4_.^23^ Although the varying amounts of water led to different shapes, the exact contribution of water towards the tuning of various morphologies is yet to be fully understood. Similarly, Chirea et al. synthesized gold nanowire networks by reducing HAuCl_4_ with sodium borohydride in reline at 40 °C.^20^ It was shown that both urea and chloride species are present on the surface of the gold nanowire network, stabilizing the nanostructure formed by fast reduction of Au^3+^ by sodium borohydride due to the formation of an intermediate adduct between choline chloride, urea, and AuCl_4_^–^. In addition to this, another study has reported the synthesis of gold nanostructures in a DES formed by a mixture of choline chloride, gallic acid, and glycerol, where the carboxyl and hydroxyl groups present in gallic acid act as the reducing and stabilizing agents, respectively, to synthesize gold nanostructures.^24^

In this paper, we report the synthesis of small gold nanoparticles (<3 nm) in pure reline and reline/water systems without the use of any external additives, reducing agents, or surfactants, where the solvent itself fulfils these multiple roles. The formation of gold chloro-complexes in reline leads to stabilization of the Au^+^ species and an easier reduction of gold metals in comparison to the species formed in water/reline mixtures. The reducing action is believed to be associated with the formation of ammonia through the hydrolysis of urea in reline at high temperatures. However, in the presence of reline, the reduction reaction is considerably faster in comparison to the individual components due to the different speciation mentioned above. Reline is also capable of acting as a weak stabilizer by partially stabilizing monodisperse gold nanoparticles during the reduction. This work provides a unique mechanistic understanding of the reduction of metal salts in deep eutectic solvents, paving the way for future green routes for the synthesis of metal nanoparticles using nontoxic, biodegradable components with minimal waste.

## Experimental Methods

The deep eutectic solvent reline was prepared by mixing choline chloride (>98% pure, Alfa Aesar) and urea (Acros Organic) in the molar ratio of 1:2, respectively, in a fumehood. Both solids were stirred for 3 h at 80 °C until a liquid was formed.^14^

In a typical gold nanoparticle synthesis, 10 mL of freshly prepared reline (or reline:water mixture (1:10 molar ratio, respectively)) was heated in an oil bath to the desired reaction temperature (30, 60, 100, and 140 °C) under stirring. Once the temperature was achieved, 10 μL of an aqueous 30 wt % HAuCl_4_ solution (Sigma Aldrich) precursor was added, leading to a 1.445 mM gold concentration. The reaction was left to proceed under constant stirring for a number of hours depending on the reaction temperature while visually monitoring the change of color. After the synthesis, the reaction was quenched by quickly dropping the temperature in an ice/water bath.

Identification of the gases released during the gold nanoparticle synthesis was carried out using an Agilent gas chromatograph equipped with a mass spectrometer detector and a DB-WAX column. For this, 250 μL of the gas vapors released by heating pure reline and reline/water mixtures was collected with a glass syringe and directly injected in the GC. ^13^C NMR and DEPT-135 (Distortionless Enhancement by Polarization Transfer) were carried out using a 400 MHz Avance III HD Smart Probe spectrometer. For sample preparation, 750 μL of the samples was diluted with 750 μL of D_2_O prior to analysis.

The synthesized gold nanoparticles were characterized using high resolution-transmission electron microscopy (HR-TEM). In order to avoid post-synthesis agglomeration, the colloidal solutions were mixed with a solution containing bovine serum albumin (BSA) protein following a reported protocol.^25^ In order to remove any remnants of reline, the samples were washed thoroughly with ethanol and water alternatively prior to drop-casting the solution over a holey carbon film on a copper TEM grid. Standard transmission electron microscopy images of the samples were acquired using a FEI Tecnai F20 G2 200 kV FEGTEM with a Gatan image filter (GIF) 200 followed by a 4 k × 4 k CCD detector. Aberration-corrected scanning transmission electron microscopy (AC-STEM) was carried out on a probe-corrected JEOL ARM200F operated at 200 kV at the electron Physical Science Imaging Centre (ePSIC). A probe convergence semiangle of 24 mrad and a probe current of 13 pA were used for the simultaneous acquisition of annular dark-field (ADF) and bright-field (BF) images. For ADF, an inner collection semiangle of 55 mrad and an outer collection semiangle of 215 mrad were used, while BF images were recorded with a collection semiangle of 15 mrad. The particle size distributions were calculated using the ImageJ software and the particle sizes were expressed as average ± standard deviation. Several micrographs were taken from different areas of the TEM grid to calculate the lateral size diameter of the particles.

X-ray photoelectron spectroscopy (XPS) was used to determine the different oxidation states of gold using a Thermo Scientific ESCALAB 250 Xi Spectrometer equipped with a monochromated Al K α source operating at 13.5 kV and 0.0205 mA. The pressure during the XPS analysis was between 10^–7^ and 10^–9^ mbar. The samples were prepared by dissolving HAuCl_4_ in pure reline (4.82 mM) and heating the solution at 140 °C. The reaction was stopped at different time intervals when a characteristic change of color was observed (4 and 11 min, respectively). Thin layers of these samples were spin-coated on Si wafers. To ensure a homogeneous film, the substrate holder was rotated continuously at 0–5000 rpm over 2 s, at 5000 rpm for 60 s, and at 5000 to 0 rpm over 0.4 s after drop-casting 1.5 mL of the samples on the Si wafers. The wafers were dried overnight in a vacuum oven at room temperature prior to XPS analysis. Three different areas (approx. 900 μm × 900 μm) were analyzed for each sample. High-resolution gold spectra were acquired using a pass energy of 50 eV and a step size of 0.1 eV. All of the binding energies were corrected using the Au4f_7/2_ peak at 84 eV. The data were processed using the Thermo Avantage software package.

Cyclic voltammograms were obtained using a potentiostat (BioLogic VSP) connected to a three-electrode electrochemical setup with a glassy carbon rod as the working electrode (Alfa Aesar, 3.14 mm^2^ active area). Platinum wires were used as counter and reference electrodes (Alfa Aesar, 99.997% metal basis). Before the beginning of each experiment, the glassy carbon electrode was cleaned with fresh aqua regia, rinsed with ultrapure water, and subsequently washed with isopropanol and ultrapure water in a sonication bath. The platinum electrodes were cleaned with fresh aqua regia and rinsed with copious amounts of ultrapure water every time prior to use. The cyclic voltammograms were performed at different temperatures (30 °C, 100 °C, and 140 °C) using an oil bath.

Small-angle X-ray scattering (SAXS) measurements were carried out using the XENOCS Nano-inXider SAXS/WAXS system in the Materials Characterization Laboratory at ISIS Neutron & Muon source and the Anton Paar SAXS/WAXS/GISAXS SAXSpoint 2.0 system at the University of Bath. Both use a Cu K-α source with 1.54 Å wavelength, giving a q-range of 0.004 Å^–1^ < *Q* < 0.4 Å^–1^. Samples were held in glass X-ray capillaries, and a background of the pure solvent was subtracted from the data before fitting. Analysis of the SAXS data was done using NCNR Analysis Macros in Igor Pro. The scattering pattern was fitted to a spherical form factor with radius *R* and polydispersity σ.

## Results and Discussion

### Synthesis of Gold Nanoparticles in Pure Reline

**Figure 1:**
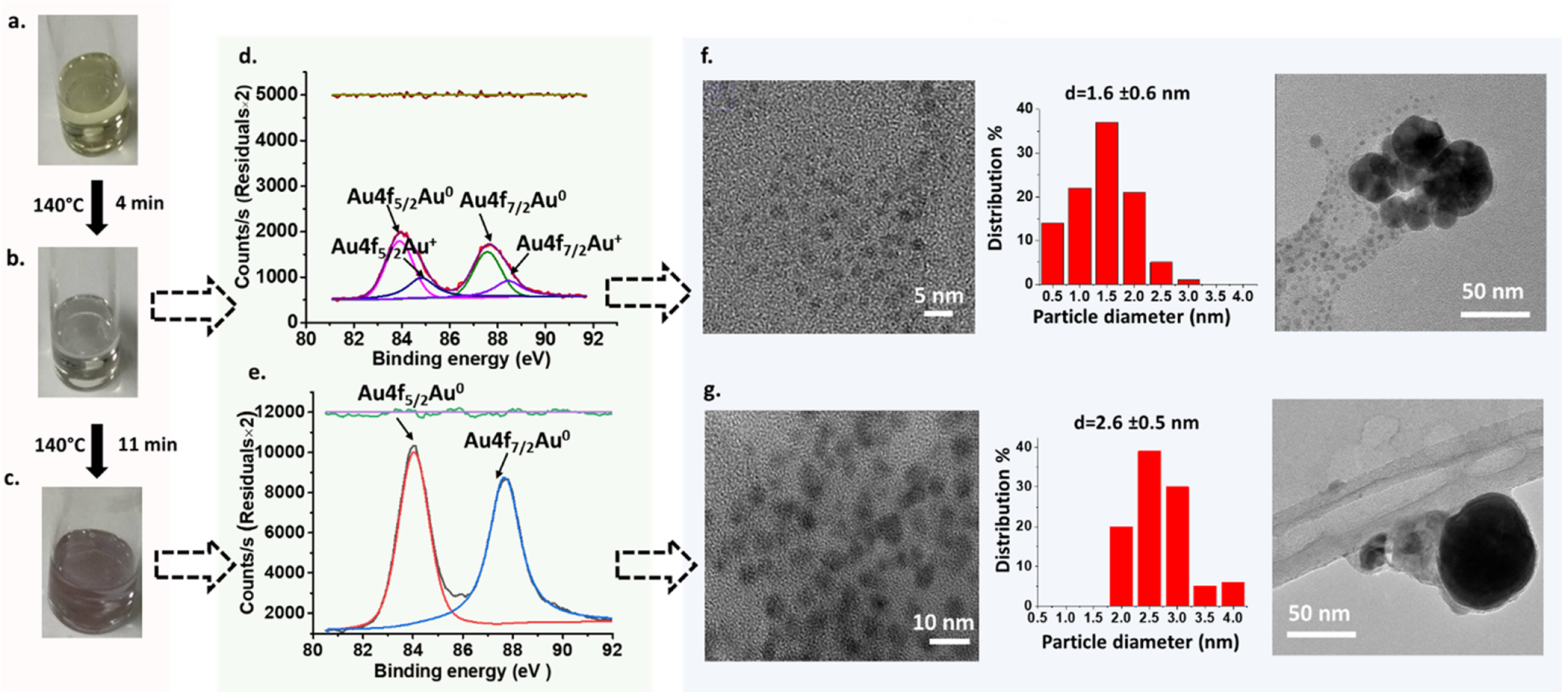
Synthesis of gold nanoparticles by reduction of HAuCl4 in pure reline: (a) yellow solution at the start of the experiment; (b) colorless solution after 4 min; and (c) ruby red solution after 11 min. XPS spectra of (d) colorless solutions and (e) ruby red solution. HR-TEM images and corresponding size histograms of (f) colorless solution and (g) ruby red solution. Additional pictures with smaller magnifications show the presence of larger aggregates not included in the histograms. Reaction conditions: initial HAuCl4 concentration: 1.445 mM, 140 °C; the colorless sample was quenched in ice-cold water after 4 min and the ruby red color solution was quenched in ice-cold water after 11 min.

The synthesis of gold nanoparticles using HAuCl_4_ as the precursor was carried out in pure reline (1:2 molar mixture of choline chloride and urea) at 140 °C under continued magnetic stirring. As the reaction proceeded, the initial yellow color of the precursor (Au^3+^) turned to colorless after ∼4 min followed by a ruby red color after 11 min (Figure 1a–c) in a clear stepwise manner. Further stirring of the solution led to a ruby red color precipitate and a clear supernatant. HR-TEM confirmed the presence of gold nanoparticles in both the colorless and ruby color solutions. In the case of the colorless sample, gold nanoparticles showed a narrow size distribution of very small particles (1.6 ± 0.6 nm, Figure 1f). Previous studies have reported that colloidal suspensions of gold nanoparticles with sizes <2 nm are colorless,^26^ in agreement with our observations. On the other hand, slightly bigger particles with similar size distribution (2.6 ± 0.5 nm, Figure 1g) were observed in the ruby color suspension. It is important to notice that, in both cases, a small number of large agglomerates (∼ 50 nm) were present, as shown in Figure 1f,g.

To evaluate the level of conversion of the gold precursor in both cases, the oxidation state of the gold species in both solutions was evaluated using X-ray photoelectron spectroscopy (XPS) after quenching the synthesis in ice-cold water. The XPS spectrum of the colorless solution (Figure 1d) showed a doublet at 84.9 and 88.4 eV corresponding to Au4f_5/2_ and Au4f_7/2_, respectively, indicative of Au^+^, whereas the doublet at 84.0 and 87.7 eV is associated with Au4f_5/2_ and Au4f_7/2_, respectively, indicative of Au^0^ presence.^26,27^ The mixture of cationic Au^+^ and Au^0^ indicates the reduction of all Au^3+^ without its full conversion to gold nanoparticles and without the disproportionation reaction 1 taking place.^10^
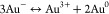
1In contrast, the XPS spectrum of the ruby red solution (Figure 1e) showed doublets for only Au4f_5/2_ and Au4f_7/2_ at 84.0 and 87.7 eV, respectively, associated with Au^0^, suggesting the full reduction of the Au^3+^ precursor into Au^0^ in just 11 min.

**Figure 2:**
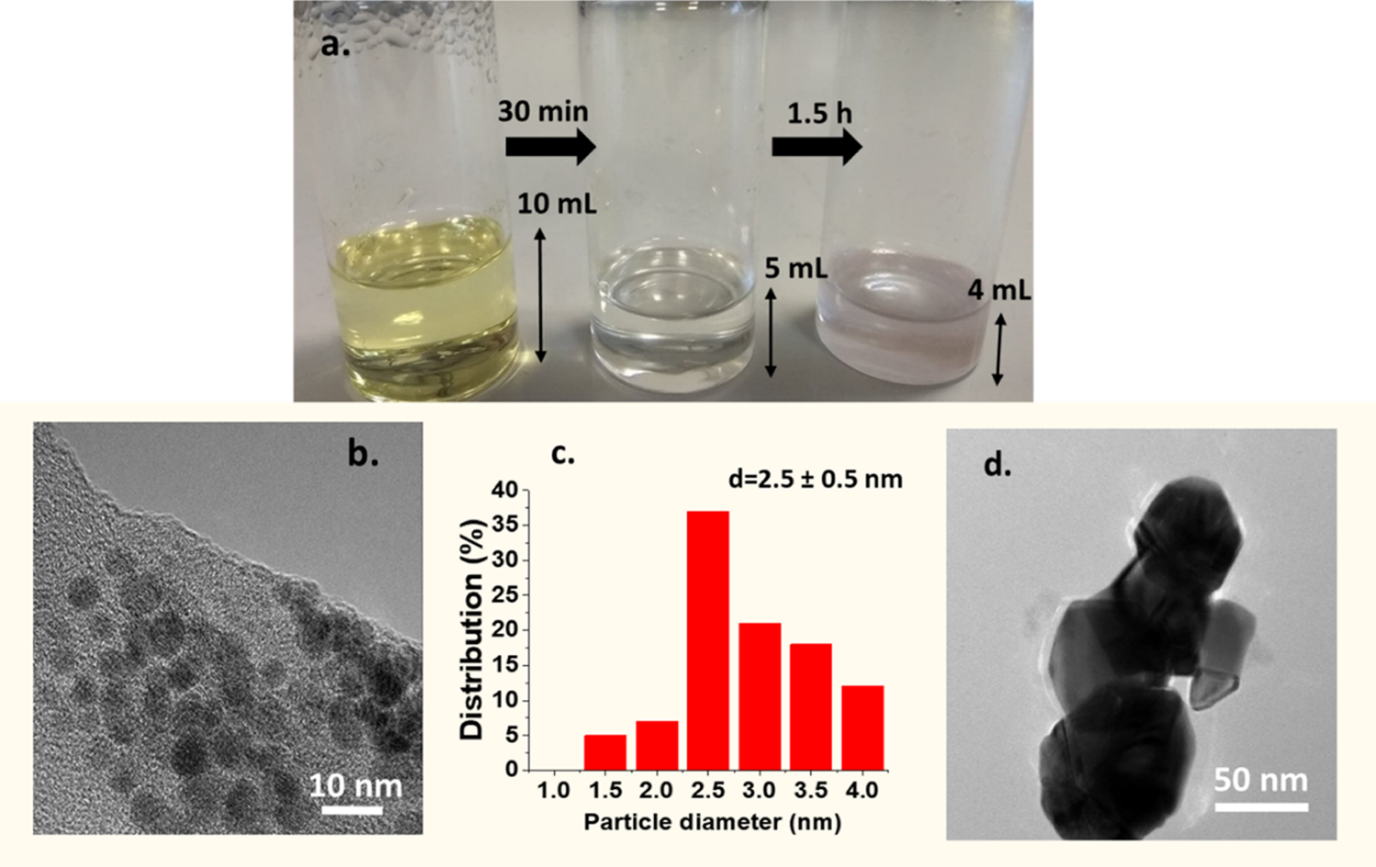
Synthesis of gold nanoparticles by reduction of HAuCl4 in reline/water (1:10 molar ratio) mixture (a). Stepwise change of color of the solution with time. (b) HR-TEM images of the ruby red solution after a 1.5 h reaction. (c) Corresponding size of the histogram; (d) scattered agglomerates. Reaction conditions: initial HAuCl4 concentration: 1.445 mM, 140 °C; the colorless sample was quenched in ice-cold water after 30 min and the ruby red color solution was quenched in ice-cold water after 1.5 h.

A similar gold nanoparticle synthesis was carried out, but this time using a mixture of reline/water (1:10 molar ratio) instead of pure reline, at the same temperature of 140 °C. A similar stepwise change of color from the initial yellow color of the precursor (Au^3+^) to colorless to ruby red was observed; however, in this case it took ∼30 min and 1.5 h, respectively, instead of a few minutes in the case of pure reline (Figure 2a). Thus, the reduction rate was considerably slower in reline/water mixtures compared to pure reline, partially due to the lower reaction temperature due to the water evaporation, as evidenced by the condensation of water droplets on the walls of the reactor vials. HR-TEM pictures showed that after 1.5 h, the ruby red suspension contained gold nanoparticles with an average size of 2.5 ± 0.5 nm (Figure 2b,c) along with scattered agglomerates of ∼50 nm (Figure 2d), which have a similar size to the particles formed in pure reline.

**Figure 3:**
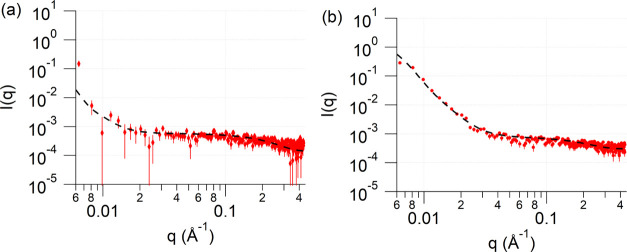
SAXS of 2.89 mM HAuCl4 solution in pure reline heated at 140 °C after (a) 4 min (colorless) and (b) 11 min (ruby red). The samples were quenched using ice-cold water.

In order to elucidate whether the observed agglomerates were formed during the synthesis of nanoparticles or during post-synthesis agglomeration in the absence of stabilizing agents, *in situ* small-angle X-ray scattering (SAXS) measurements of the solutions were conducted. A series of samples were prepared by dissolving HAuCl_4_ (1.445 mM and 2.89 mM) in pure reline and heating the solution at 140 °C. The reaction was quenched at 4 and 11 min to replicate the conditions described above. Similarly, a gold precursor concentration of 1.445 mM was used to mimic the conditions above (Figure S1), while a solution containing double the concentration of the initial gold precursor of 2.89 mM was needed to increase the signal to noise ratio to ensure the scattering of small nanoparticles (∼1 nm) could be seen (Figure 3).

The data from the colorless solution after 4 min of heating (Figure 2a) showed a bimodal distribution of nanoparticles: one with an average radius of 0.9 ± 0.1 nm and polydispersity of 11%, and the other with a radius of 49.5 ± 10.5 nm and polydispersity of 25%, in agreement with the HR-TEM images (Figure 1f). Similarly, the data after 11 min of heating (ruby red suspension) indicated the presence of both small nanoparticles with a radius of 1.0 ± 0.02 nm and a polydispersity of 12% and bigger agglomerates with a radius 31.1 ± 0.5 nm and polydispersity of 33% (in agreement with HR-TEM, Figure 1g). These data indicate that the agglomeration of nanoparticles takes place during their synthesis, suggesting a weak stabilization by the deep eutectic solvent reline. It was not possible to perform similar SAXS measurements when using reline/water mixtures because of the problems of aggregation and sedimentation of gold particulates, which suggests that similar agglomeration during synthesis takes place in the reline/water mixtures.

**Figure 4:**
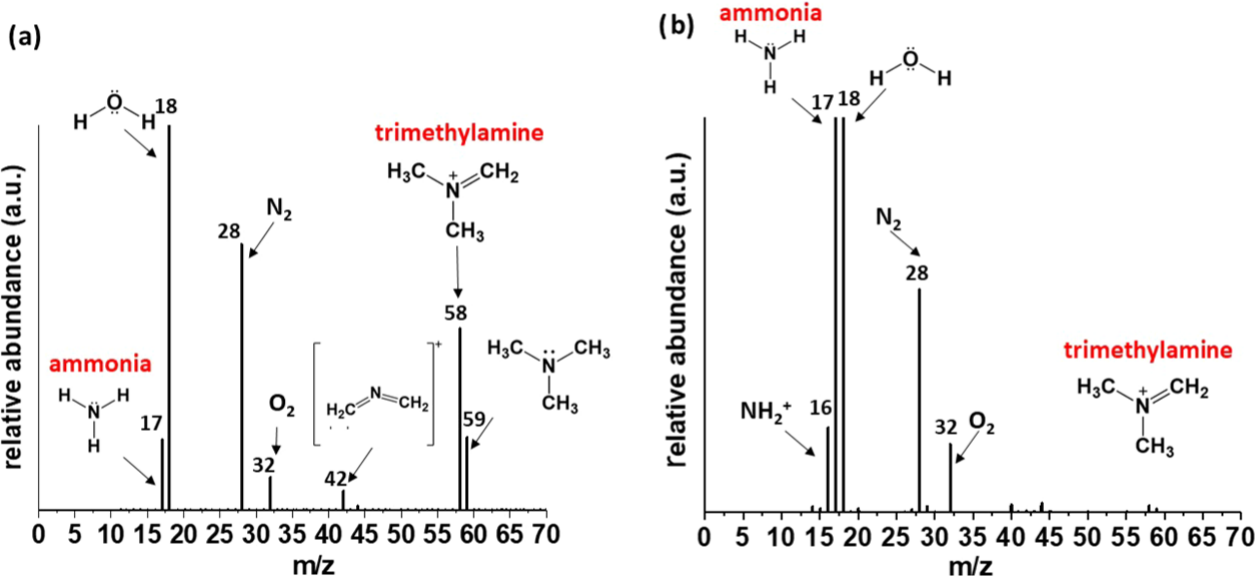
GC-MS spectra of gases released during the synthesis of gold nanoparticles in (a) pure reline and (b) reline/water mixture of 1:10 molar ratio at 140 °C.

To understand the mechanism of formation of gold nanoparticles and the nature of the reducing agents in the reline and reline/water mixture, experiments were performed to identify the gases released during the high-temperature synthesis. A damp litmus test confirmed the release of alkaline gases in both cases, which were identified by GC-MS. The GC-MS spectrum of the gas sample taken during the synthesis in pure reline showed typical peaks for trimethylamine (*m*/*z* = 59), [CH_2_=N=CH_2_]^+^ (*m*/*z* = 42),^28^ [(CH_3_)_2_N(CH_2_)]^+^^29^ (*m*/*z* = 58), and traces of ammonia (*m*/*z* = 17) and water vapor (*m*/*z* = 18) in addition to nitrogen (*m*/*z* = 28) and oxygen (*m*/*z* = 32), as shown in Figure 4a. The counterpart GC-MS spectrum of the gas sample taken during the synthesis in reline/water mixtures at 140 °C showed the predominant presence of ammonia (m/z = 17), water vapor and NH_4_^+^(*m*/*z* = 18), N_2_ (*m*/*z* = 28), and O_2_ (*m*/*z* = 32) (Figure 4b).

Ammonia was released from both pure reline and reline/water mixtures due to urea hydrolysis (2). In the case of pure reline, traces of water present due to its hygroscopic nature as well as the aqueous solution of the gold precursor are believed to be responsible for the ammonia formation. When reline components (choline chloride and urea) were dried prior to the formation of reline, a similar color transition was observed, but slightly delayed on time.
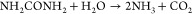
2The lack of CO_2_ in the GC-MS spectra might suggest that initially only the first urea hydrolysis step (3) takes place with H_2_N-COOH remaining in the solution
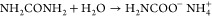
3The trimethylamine observed during the synthesis in pure reline is formed by decomposition of choline chloride^30^ through the SN2 Reaction 2. In pure reline, there is a strong interaction between the choline chloride ion pair favoring this reaction. In contrast, in reline/water mixtures, the water molecules will shield the electric charges, reducing the chloride nucleophilicity and disfavoring the reaction.
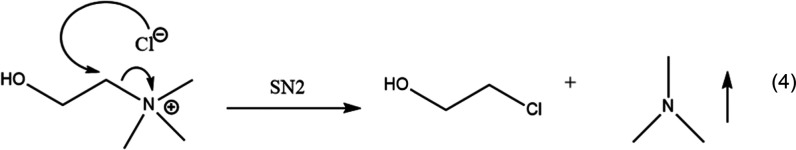
4

Control experiments with an aqueous solution of choline chloride (without any urea) at 140 °C did not show any change in color from the initial pale yellow of the gold precursor, suggesting no formation of gold nanoparticles. This observation indicated that neither choline chloride nor its thermal decomposition product, trimethylamine, was responsible for the reduction of gold and thus the formation of nanoparticles. The absence of N-H bonds in trimethylamine makes it a weaker reducing agent than ammonia.^31^ However, when the gold precursor was added to an aqueous solution of urea (with no choline chloride) prepared in the same ratio as reline (but replacing choline chloride with water in 1:2 water:urea mixture), gold nanoparticle formation was observed within 1.5 h after the solution was heated at 140 °C, evidenced by the color change from the pale yellow of the gold precursor to ruby red, indicating that urea and/or ammonia was responsible for the reduction of gold precursor and thus the formation of nanoparticles. It is important to highlight that the rate of reaction in this case is similar to the one observed above when using reline:water 1:10 molar ratio mixtures. Equivalent control experiments in aqueous ammonia solutions (1, 5, and 28 wt %) failed due to the fast evaporation of ammonia when heating the solutions in a bath at 140 °C.

To further substantiate the hypothesis of in situ formed ammonia being responsible for the reduction of gold, two experiments under identical conditions were carried out in the DES formed by choline chloride/glycerol (1:2 molar ratio) and glycerol/urea (1:1 molar ratio). In the former experiment, no formation of gold nanoparticles was observed, while the yellow-transparent-purple color transition was observed in the latter case within ∼1 h.

Two additional experiments were carried out, one in aqueous urea solution and the other in pure reline, but in this case, at 30 °C, the temperature at which the hydrolysis of urea to form ammonia does not take place.^32^ Under these conditions, no gold nanoparticles were formed in the aqueous urea solution, as indicated by the constant yellow color of the gold precursor. However, during the synthesis in pure reline at room temperature, the solution turned to colorless after being stirred for three days. Colorimetric studies based on Berthelot’s reaction^33^ (Figure S2) showed the presence of ammonium ion in pure reline, likely formed during its preparation when the mixture of choline chloride and urea solids was heated to 80 °C. These control experiments suggest that indeed, ammonium ions from the hydrolysis of urea are responsible for the reduction of gold to form nanoparticles in urea solutions, reline/water mixtures, and pure reline. The reducing potential of ammonia is associated with the lone pair of electrons on the nitrogen atom available for donation, and it has been widely used as a reducing agent to synthesize ZnO, CoFe_2_O_4_, and silver nanoparticles.^32^ In this latter case, ammonia forms Ag[NH_3_]_2_^+^ complexes that deplete the availability of ionic silver in the medium, resulting in quasi-monodisperse silver nanoparticles.^34,35^

Although these experiments revealed that the formation of gold nanoparticles is associated with the reducing nature of ammonia formed by the hydrolysis of urea, it is key to highlight that the reduction is considerably quicker in pure reline compared to aqueous urea solutions (a few minutes *versus* 1.5 h, respectively) despite the fact that urea hydrolysis is favored in the presence of water, as shown in GC analyses of the gases (Figure 4). Such observations reveal the role of choline chloride-based DES in the speciation of the gold precursor with the formation of gold chloro-complexes (e.g., AuCl_4_^–^) by the high chloride activity of the reline, as previously reported for Au and Cu in choline-chloride-based DES and highly concentrated aqueous chloride solutions, stabilizing monovalent ions.^36^ The possibility of choline acting as a ligand forming Au(choline)Cl_3_^–^ species or similar has also been reported.^37^ A different speciation is expected in the presence of large amounts of water, such as in the reline/water mixture studied here, where the labile chloride ligands are replaced by water molecules, leading preferably to the formation of Au(H_2_O)_3_ species.

**Figure 5:**
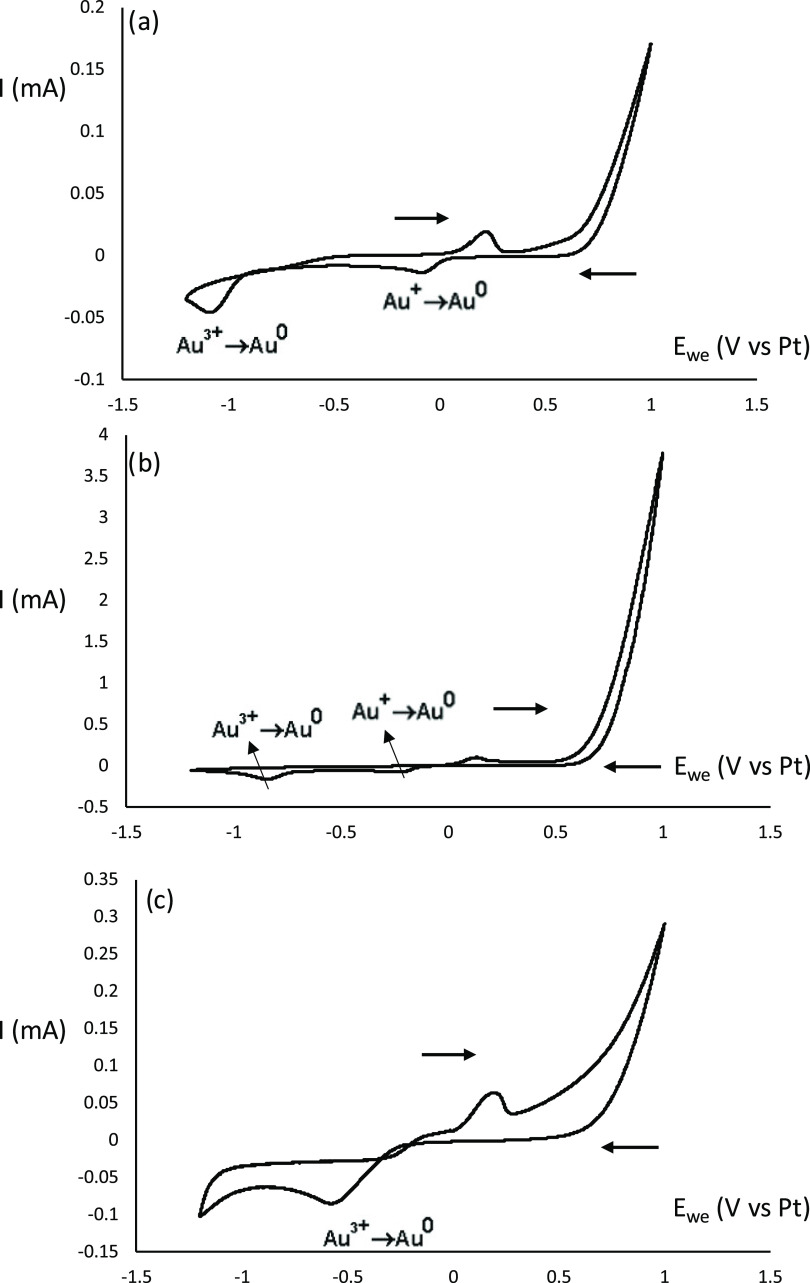
Cyclic voltammogram of HAuCl4 in (a) pure reline, (b) reline/water (1:10 molar ratio), and (c) pure water at 30 °C. Conditions: 10 mL of pure reline or reline/water (1:10 molar ratio), HAuCl4 (2.25 mM), 20 μg of AuCl, and 2 mL of as-prepared gold nanoparticles were added. All of the three gold standards were added (Au0, Au+, and Au3+) to ensure that all species are present in reline for proper peak identification.

Electrochemical measurements confirm the different speciation as well as the stabilization of Au^+^ species in pure reline. Figure 5 shows the cyclic voltammogram of the HAuCl_4_ precursor in pure reline, reline/water 1:10 mixture, and pure water. In pure reline, two reduction peaks corresponding to the reduction of Au^3+^ and Au^+^, respectively, are observed. However, in the presence of water, a single reduction peak associated with the reduction of Au^3+^ is observed due to the lack of stability of Au^+^, which rapidly disproportionates to Au^3+^ and Au^0^ (Reaction 1).^38^ It is only in the presence of pure reline that Au^+^ species is stabilized. In addition, one should note that the reduction of Au^+^ lies in the positive potential range, as it is well known that the speciation of metal complexes has a strong influence on their redox potential.^15,39^

These electrochemical studies also showed that upon increasing the temperature, the reduction potentials were shifted to more anodic values for both pure reline and reline/water 1:10 molar ratio systems as the reduction is favored. For example, with an increase in temperature, the reduction potential shifts from −1.08 V at 30 °C to −0.71 V at 140 °C for pure reline. The fact that higher temperatures can be achieved in pure reline than in water/reline mixtures further confirms the higher reduction rate, and consequently gold nanoparticle synthesis, observed.

**Figure 6:**
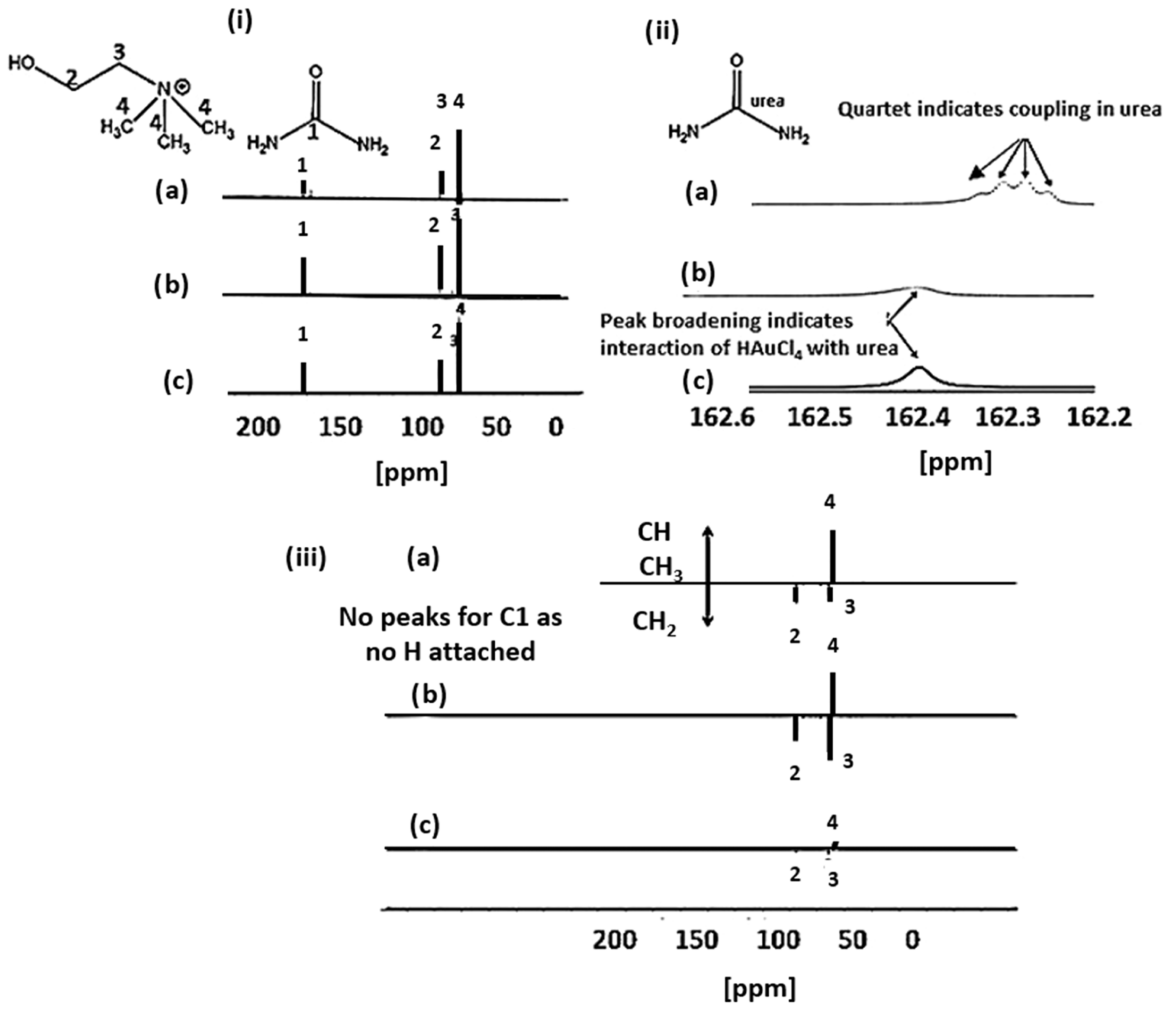
(i) ^13^C NMR spectra; (ii) urea decoupling shown in ^13^C NMR spectra (zoomed view of part (i)); (iii) DEPT-135 spectra in (a) pure reline, (b) HAuCl_4_/reline mixture, and (c) supernatant left after the precipitation of gold nanoparticles at 140 °C (within 2–3 h of HAuCl_4_ addition). Initial HAuCl_4_ concentration: 1.445 mM.

To explore the potential change of reline during the reduction and gold nanoparticle formation, ^13^C NMR and DEPT-135 analyses (Figure 6) were carried out. The ^13^C NMR spectra of pure reline showed peaks associated with urea at 162.21 ppm, α-CH_2_ at 68.20 ppm, β-CH_2_ at 56.28 ppm, and Me_3_ at 54.40 ppm associated with choline, denoted as 1, 2, 3, and 4, respectively. In addition, Figure 6ii shows a zoom of the urea decoupling area. Before adding the gold precursor, a quartet was seen at 162.21 ppm in the pure reline corresponding to the coupling of the carbon to the two nitrogen atoms in the urea molecule. Theoretically a quintet 1:2:3:2:1 would have been expected; however, a quartet is observed because of the low coupling (^13^C-^14^N) constant. The spectra show the lack of any considerable structural rearrangement in reline during the synthesis of gold nanoparticles, such as oxidation of the alcoholic group in choline chloride to an aldehyde. This lack of structural changes is further evidenced by distortionless enhanced polarization transfer (DEPT-135) measurements, where the multiplicity of the carbon atoms was determined using a 135-degree decoupler pulse. The DEPT-135 spectra were phased to show positive CH_3_ and CH signals and negative CH_2_ signals (Figure 6iii). No changes in the spectra were observed after addition of the gold precursor or after the gold nanoparticle synthesis at 140 °C. The slight downfield shift in the ^13^C NMR spectra observed in all of the peaks after addition of the acidic gold precursor (HAuCl_4_) is caused by the decrease of pH, which was confirmed by a control experiment where an equivalent amount of HCl was added.

Reline plays a clear role, albeit weak, in the stabilization of the formed gold nanoparticles. A similar urea-based stabilization of gold nanowire networks has been previously reported in reline due to the formation of an intermediate adduct with the formula [HO-CH_2_-CH_2_-N^+^(CH_3_)_3_]AuCl_4_^–^·2(NH_2_)_2_CO between choline chloride, urea, and AuCl_4_^–^.^20^

**Figure 7:**
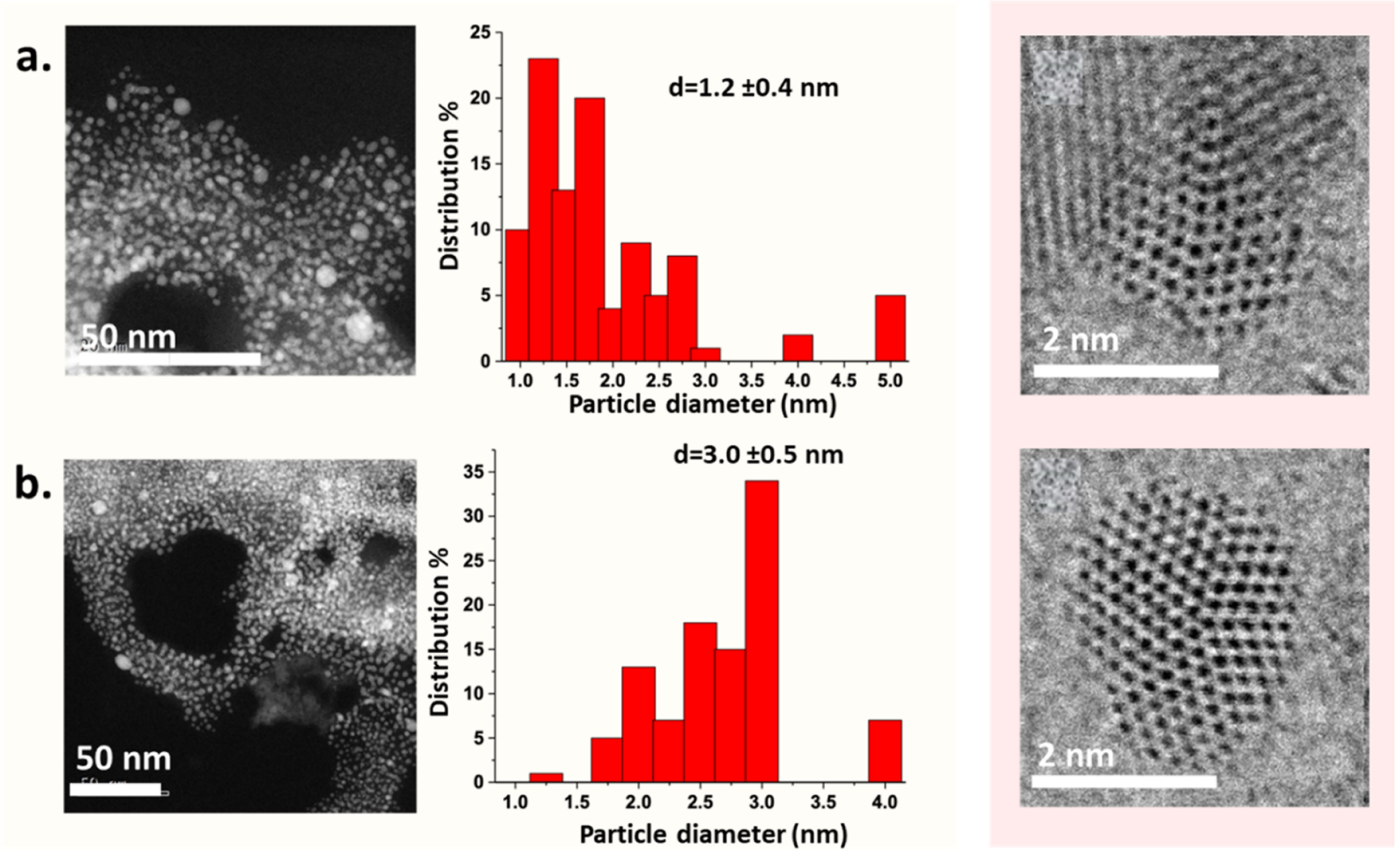
ADF STEM representative images and corresponding particle size histogram of (a) colorless solution after 4 min and (b) ruby red solution after 11 min. Reaction conditions: initial HAuCl4 concentration: 1.445 mM, PVP: 0.05 wt %, 140 °C; the colorless sample was quenched in ice-cold water after 4 min and the ruby red color solution was quenched in ice-cold water after 11 min. High-resolution BF STEM images show decahedral particles oriented along the <110> zone axis. All STEM micrographs were acquired at a pixel dwell time of 20 us/pixel with 1024 × 1024 pixels.

A better stabilization of the gold nanoparticles can be easily achieved by the addition of poly(vinylpyrrolidone) (PVP) during the synthesis of the gold nanoparticles in pure reline. Figure 7a shows that very small gold nanoparticles with a narrow distribution (1.2 ± 0.4 nm) are obtained in a few minutes. Similarly, the size increases slightly after complete gold reduction in the ruby red solution (3.0 ± 0.5 nm). Both average sizes are similar to the ones in the absence of PVP, suggesting that the presence of the polymer does not interfere in the reduction reaction. High-resolution AC-STEM shows the decahedral structure of the gold nanoparticles and also reveals the presence of scattered, very small clusters in both solutions (Figure S4).

Despite that, the interaction of reline/water and gold nanoparticles is expected to be considerably different from the case of pure reline because of the considerably slower reduction rate in the former case (1.5 h *versus* few minutes, respectively). Such difference is attributed to the loss of the hydrogen-bonding framework in the reline in the presence of water. Several studies have shown that the water present in reline beyond a certain concentration (∼25 to 41 wt %) disrupts the intermolecular hydrogen-bonding framework and solubilizes the parent components in reline.^40,41^ In our case, the 1:10 reline:water molar ratio corresponds to 45 wt % water, and such a system is best described as an aqueous solution of DES38.^42^ Under these conditions, it is likely that the activation of the gold precursor does not take place while the disproportionation reaction does occur, decreasing the rate of reduction despite ammonia being identified in both cases as the reduction agent.

## Conclusions

A mechanistic understanding of the synthesis of gold nanoparticles in the environmentally friendly deep eutectic solvent reline (formed by choline chloride and urea) shows the stepwise reduction from Au^3+^ → Au^+^ → Au^0^ without addition of any external reducing agents. Indeed, we demonstrate that ammonia, formed by hydrolysis of the urea component, acts as a reducing agent. Neither choline chloride nor its decomposition product trimethylamine plays a role as a reducing agent. The reduction rate of gold is very fast in pure reline (full reduction within a few minutes) in comparison to aqueous solutions of reline or aqueous urea, which required ∼1.5 h, despite the considerably lower concentration of ammonia in pure reline. This is attributed to the different speciation of the gold precursor in both environments, with the formation of gold chloro-complexes in pure reline with a strong influence on the redox potential due to the labile nature of chloride. However, in the presence of water, water replaces chloride ligands in the metal complexes with an associated shift in redox potential. This work also shows the stabilization of the gold nanoparticles in the presence of reline (even in the presence of water). Such stabilization leads to the synthesis of small particles (<3 nm) with narrow size distributions. However, the presence of scattered agglomerates suggests the weak nature of the stabilization. Indeed, in the presence of stabilizers such as PVP, no agglomerates are observed. This work presents a novel green route for the synthesis of small metal nanoparticles in the absence of any external additives.

## Supporting Information Available

SAXS data, protocol for the Barthelot’s reaction and supplementary high-resolution STEM ADF pictures (PDF)
