# Supplementary material for: Role of the Deep Eutectic Solvent Reline in the Synthesis of Gold Nanoparticles
Source: ACS Sustain Chem Eng. 2023 Jul 3;11(28):10242–51. doi: 10.1021/acssuschemeng.2c07337 (PMC10354802; doi:10.1021/acssuschemeng.2c07337)
Supplement: Supplementary file 1 — sc2c07337_si_001.pdf [file sc2c07337_si_001.pdf]

The role of deep eutectic solvent reline in the synthesis of gold nanoparticles

Sukanya Datta[1], Julien Mahin[1], Emanuela Liberti[2,3], Iva Manasi[4], Karen J. Edler[4], Laura Torrente-Murciano[1]*

[1] Department of Chemical Engineering and Biotechnology, University of Cambridge, Philippa Fawcett Drive, CB3 0AS, Cambridge, UK

[2]Department of Materials, University of Oxford, OX1 3PH, Oxford UK
[3]The Rosalind Franklin Institute, Harwell Science & Innovation Campus, Didcot, OX11 0QS, Oxfordshire, UK
[4]Department of Chemistry, University of Bath, Claverton Down Road, BA2 7AY, Bath, UK

Supporting information

SAXS data (1.445 mM $HAuCl_4$)

When the initial $HAuCl_4$ concentration in the solution is 1.445 mM, nanoparticle agglomerates with a radius of 27.8 ± 1.6 nm and polydispersity of 28% are observed for the ruby red Au^0 solution Figure S1a). However in the case of colourless Au^+ solution, it is noted that the signal to noise ratio is too weak and hence any scattering from ~1 nm particles is masked easily (FigureS1b).

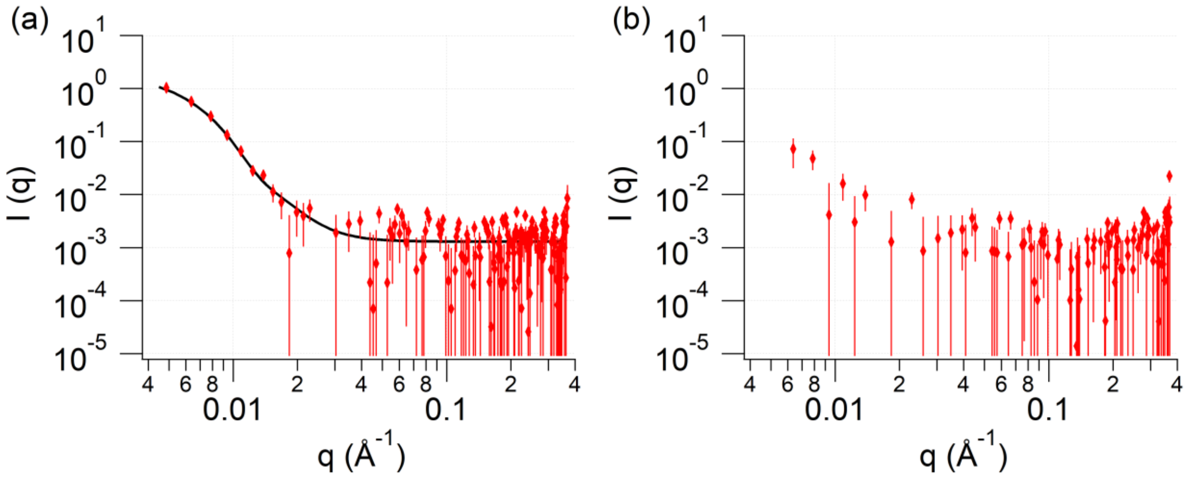

***Figure S1**: SAXS from Au^0 (ruby red) and Au^+ (colourless) solutions in pure reline. (a) SAXS from Au^0 solution with initial concentration of $HAuCl_4$ at 1.445mM; (b) SAXS from Au^+ solutions with initial concentration of $HAuCl_4$ at 1.445 mM*

[1] Corresponding author: Prof Laura Torrente-Murciano, lt416@cam.ac.uk

Berthelot's reaction

Colorimetric studies show the presence of ammonium ion in pure reline that explains the reduction of gold without heating reline during the synthesis of gold nanoparticles. The commercial colorimetry kit purchased from Merck uses the Berthelot's reaction to detect ammonium ions in the solution as the reaction turns blue due to the formation of indophenol upon reaction with ammonium present in the liquid as seen in eq. 4. In the Berthelot's reaction, chloramine is initially produced when the ammonium ion interacts with hypochlorite and subsequently quinonechloramine is obtained upon the reaction of chloramine with phenol. Indophenol is produced when phenol reacts with quinonechloramine (eq. 4). The absorption spectrum of indophenol blue 650 nm (Figure S2) matches with the literature[246].

During the preparation of pure reline at 80 °C, urea decomposes to produce ammonium ions in the solution phase. Another experiment is carried out by adding aqueous ammonia to the aqueous solution of urea at 30 °C in the presence of gold precursor and the solution colour changes from pale yellow to colourless within a minute indicating the reduction of Au^{3+} to Au^{+} indicating the reducing action of ammonia.

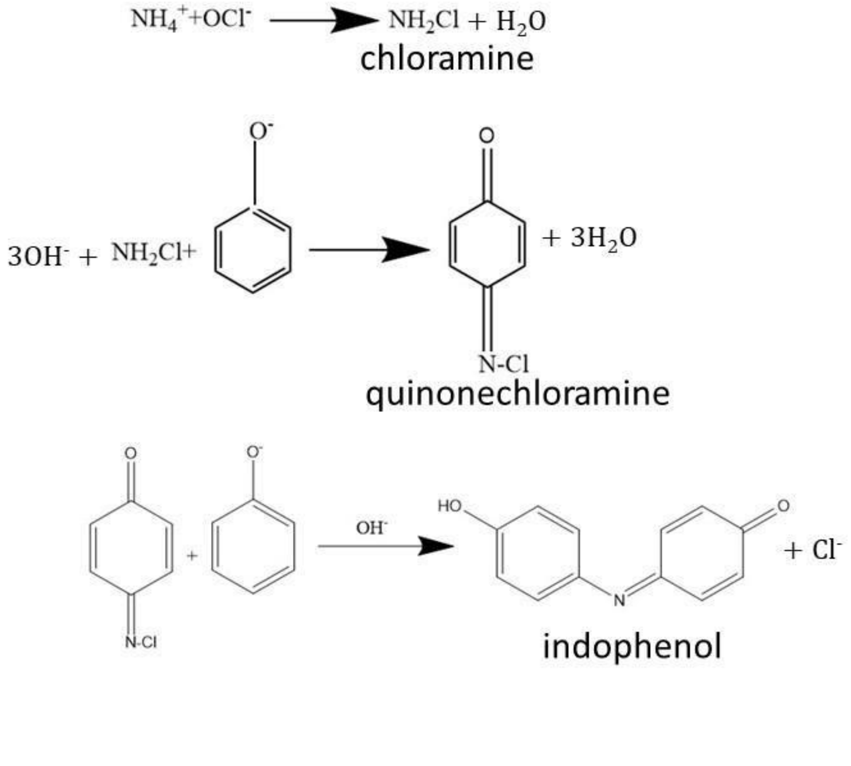

(2)

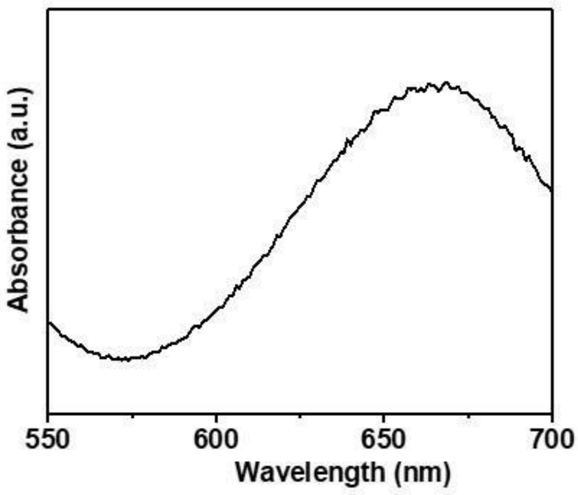

Figure S2: Absorbance spectra of indophenol formed as a result of Berthelot's reaction with ammonium ions in pure reline. Pure reline is prepared by combining choline chloride and urea in the molar ratio of 1:2 by heating at 80 ℃ for 2-3 h.

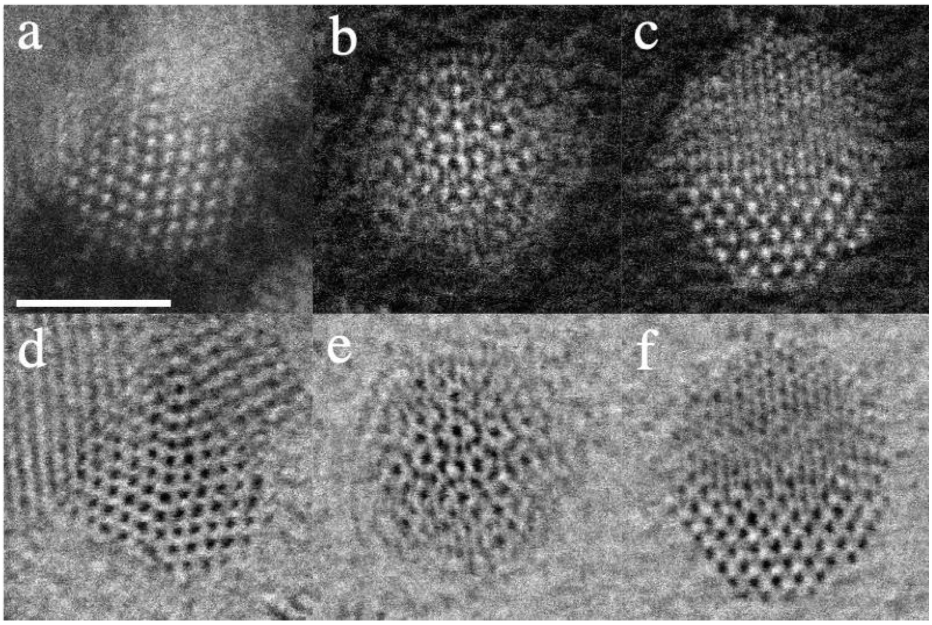

Figure S3: Simultaneously acquired high-resolution STEM ADF (a), (b), (c) and STEM BF (d), (e), (f) representative of Au nanoparticles in the red solution. Both decahedral and icosahedral face centred cubic structures are observed. In (a) a decahedral particle is aligned close to a <110> zone axis. The image contrast in this case resemble that of a particle aligned in <110> orientation and containing a single twin on a {111} plane [1]. (b) and (c) represent icosahedral and decahedral particles oriented away from a <110> zone axis. Scale bar is 2 nm. 1024 x 1024 micrographs were acquired at a pixel dwell time of 20 us/pixel.

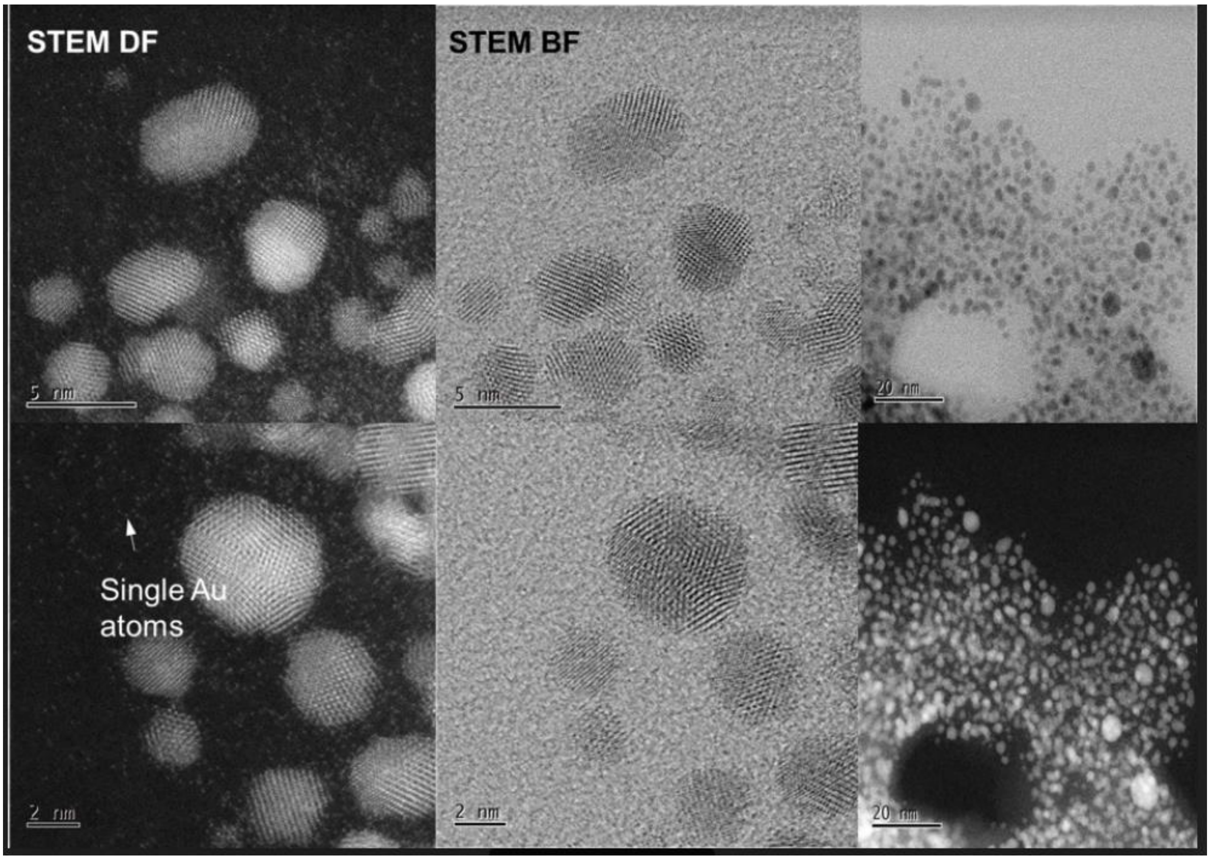

Figure S4: Single Au atoms in pure reline (red solution)
